# Amphetamine-induced dopamine release in rat: Whole-brain spatiotemporal analysis with [^11^C]raclopride and positron emission tomography

**DOI:** 10.1177/0271678X231210128

**Published:** 2023-10-26

**Authors:** Jarkko Johansson, Madelene Ericsson, Jan Axelsson, Sara af Bjerkén, Ana Virel, Nina Karalija

**Affiliations:** 1 Department of Radiation Sciences, Diagnostic Radiology, Umeå University, Umeå, Sweden; 2 Umeå Center for Functional Brain Imaging (UFBI), Umeå University, Umeå, Sweden; 3 Umeå Centre for Molecular Medicine, 8075 Umeå University, Umeå, Sweden; 4 Department of Radiation Sciences, Radiation Physics, Umeå University, Umeå, Sweden; 5 Department of Integrative Medical Biology, 8075 Umeå University, Umeå, Sweden; 6 Department of Clinical Science, Neurosciences, Umeå University, Umeå, Sweden

**Keywords:** Amphetamine, displacement, dopamine, imaging, receptor

## Abstract

Whole-brain mapping of drug effects are needed to understand the neural underpinnings of drug-related behaviors. Amphetamine administration is associated with robust increases in striatal dopamine (DA) release. Dopaminergic terminals are, however, present across several associative brain regions, which may contribute to behavioral effects of amphetamine. Yet the assessment of DA release has been restricted to a few brain regions of interest. The present work employed positron emission tomography (PET) with [^11^C]raclopride to investigate regional and temporal characteristics of amphetamine-induced DA release across twenty sessions in adult female Sprague Dawley rats. Amphetamine was injected intravenously (2 mg/kg) to cause displacement of [^11^C]raclopride binding from DA D2-like receptors, assessed using temporally sensitive pharmacokinetic PET model (lp-ntPET). We show amphetamine-induced [^11^C]raclopride displacement in the basal ganglia, and no changes following saline injections. Peak occupancy was highest in nucleus accumbens, followed by caudate-putamen and globus pallidus. Importantly, significant amphetamine-induced displacement was also observed in several extrastriatal regions, and specifically in thalamus, insula, orbitofrontal cortex, and secondary somatosensory area. For these, peak occupancy occurred later and was lower as compared to the striatum. Collectively, these findings demonstrate distinct amphetamine-induced DA responses across the brain, and that [^11^C]raclopride-PET can be employed to detect such spatiotemporal differences.

## Introduction

The addictive and neurodegenerative properties of amphetamine are clearly established.^1^ Still, studies monitoring whole-brain amphetamine-induced dopamine (DA) activity, and their temporal trajectories, are still scarce. Such knowledge is important, as it offers a more complete understanding for the cerebral underpinnings of drug-induced behavior. One approach that allows for continuous assessment of whole-brain DA activity is positron emission tomography (PET) and a radioligand that can be displaced upon elevation of endogenous DA levels. Amenability of [^11^C]raclopride – a moderate-affinity D2-like DA receptor (D2DR) antagonist – for detection of amphetamine induced DA release in the striatum is well established. However, the applicability of [^11^C]raclopride for quantification of extrastriatal displacement from D2DRs remains unclear. The overarching aim of the present work was to map the spatiotemporal profiles of amphetamine-derived DA release across the brain with PET and [^11^C]raclopride in Sprague Dawley rats. While doing so, we learn in which regions of the rat brain [^11^C]raclopride displacement can be reliably observed.

The popularity of [^11^C]raclopride relates to its beneficial properties such as good test-retest reliability,^2,3^ and sensitivity to measure competitive binding with endogenous DA.^4,5^ Being a moderate-affinity ligand, the signal-to-noise ratio for [^11^C]Raclopride has historically been deemed too low for reliable extrastriatal assessments,^6–8^ and therefore, it has mainly been used for striatal D2DR quantifications. Instead, high-affinity D2DR radioligands, with improved cortical signal-to-noise ratio, are preferred for extrastriatal measurements. Notably, high-affinity ligands have limitations, as these are not optimal for assessment of striatal D2DR assessments and endogenous DA release.^9,10^ It is therefore appropriate to reassess the applicability of a moderate-affinity ligand, such as [^11^C]raclopride, for whole-brain coverage in the rat. In fact, a growing literature now provide compelling evidence that test-retest statistics of [^11^C]raclopride are good-to-excellent for striatal as well as for extrastriatal measurements,^2,11^ and that extrastriatal [^11^C]raclopride-derived D2DR levels are coherent with D2DR levels estimated with high-affinity D2-ligands.^3,11^ Furthermore, robust associations between striatal and extrastriatal DRD2 measured using [^11^C]raclopride have been observed in cross-sectional settings,^11^ but also in terms of 5-year change.^12^ Furthermore, associations between extrastriatal [^11^C]raclopride binding potential (BP_ND_) has been linked with parameters including age^13^ and cognitive function.^14–16^ Together, these studies suggest that extrastriatal [^11^C]raclopride binding measure functionally relevant and repeatable D2DR binding, rather than noise.

Past studies have unequivocally shown an amphetamine-induced reduction in specific [^11^C]raclopride binding in the striatum, and attributed this to increased endogenous DA release.^17^ Amphetamine elevates extracellular DA levels through intracellular synaptic vesicle depletion and promotion of reverse transport through monoamine transporters such as the DA transporter (DAT),^1,18^ which is primarily expressed in the striatum. Despite low cortical DAT concentrations, *in vivo* microdialysis experiments in rodents have evidenced amphetamine-induced cortical DA release.^19^ The amphetamine-induced cortical DA release is, however, lower and may follow a different time course than striatal DA release.^19,20^ While most previous imaging studies have neglected extrastriatal [^11^C]raclopride displacement when using amphetamine, administration of other substances, e.g. methylphenidate, tetrahydrocannabiniol, and quetiapine have been associated with changes in frontal and/or temporal cortical [^11^C]raclopride binding.^7,21,22^ Moreover, food intake-related cortical [^11^C]raclopride displacement has been found to correlate with eating desire in humans.^23^

Taken together, [^11^C]raclopride-PET may constitute a suitable approach for identification and quantification of DA release across the rat brain. The present study simultaneously investigated striatal and extrastriatal amphetamine-induced DA-release using an [^11^C]raclopride displacement approach. For this purpose, we employed automatic whole-brain segmentation and a temporally sensitive pharmacokinetic model (linear parametric neurotransmitter PET; lp-ntPET),^24,25^ to depict a temporally variant responses across the brain.^26,27^ Our primary aim was to characterize the spatiotemporal patterns of amphetamine-induced DA release across the rat brain, and while doing so, assess the validity of in-scan [^11^C]raclopride technique in extrastriatal regions. We predicted a robust amphetamine-induced [^11^C]raclopride signal change in the striatum, possibly with a ventro-dorsal striatal gradient in the magnitude of DA release,^28^ and absence of change following saline injections. Furthermore, we hypothesized that [^11^C]raclopride displacement would be observed in selected extrastriatal regions, in a patterns similar to regions previously associated with drug addiction and where D2DRs are relatively abundant (e.g. prefrontal cortex and medial temporal lobe).^29^

## Materials and methods

### Animals and study design

Animal experiments were performed in accordance with the EU directive 2010/63/EU and Swedish legislation on the protection of animals used for scientific purposes and approved by the animal review board at the court of appeal of Northern Norrland in Umeå (approval number A33-2019). Experiments are reported in compliance with the ARRIVE guidelines (Animal Research: Reporting In Vivo Experiments, www.nc3rs.org.uk/arrive-guidelines). All efforts were made to minimize animal suffering, and animals were housed under 12:12 h light-dark conditions and had access to food and water *ad libitum* during the experimental time. Adult female Sprague Dawley rats (n = 14; weight 200–250 g; Charles River) were used throughout this study. The animals underwent PET and magnetic resonance imaging (MRI) under isoflurane anaesthesia (1.5–2.0% in O_2_). Temperature and breathing were monitored during the course of the experiments. Nine animals received amphetamine and five received saline (0.9% NaCl). Five of the animals were scanned twice with amphetamine rendering a total of 14 amphetamine PET scans, and one animal was scanned twice with saline rendering 6 saline PET scans.

### PET imaging

[^11^C]raclopride was synthesized on-site at Umeå University Hospital, and imaging data was acquired on a small animal PET/CT scanner (Mediso nanoScan PET/CT, Hungary). The specific activity was determined for each batch, which was used to calculate the mass (in µg) of non-labelled raclopride for each injection. The rat tail vein was cannulated, and the cannula was flushed with heparinized saline before injecting the radiotracer. The scan procedure started with a 50 kV helical CT scan covering the head and upper body, followed by a 60-minute list-mode PET acquisition commencing at injection of 12.8 ± 5.3 MBq (non-labelled raclopride mass 0.015 ± 0.016 µg, range = 0.001 – 0.06 µg) [^11^C]raclopride. List mode data was binned to 12 × 10 s, 6 × 20 s, 6 × 40 s, 6 × 80 s, 6 × 160s, 7 × 240 s frames. This was reconstructed to dynamic PET-images of voxel-size 0.4 mm using the built-in TeraTomo 3D iterative reconstruction; employing 4 iterations, 4 subsets, and correction for attenuation, scatter, and random events. At 25 min after the start of the PET scan, rats were injected intravenously with 2 mg/kg amphetamine (d-Amphetamine sulfate) or an equivalent volume of saline (0.9% sodium chloride).

### Magnetic resonance imaging

When PET scans were completed, MRI imaging was performed in each animal for the purpose of anatomical co-registration of PET images. MRI was carried out in a 9.4 T Bruker BioSpec 94/20 scanner equipped with a BG12S-HP gradient set, utilizing a 40 mm quadrupolar volume coil from Bruker and running Paravision 6 software (Bruker, Ettlingen, Germany). A T2-RARE 3D sequence was acquired (TR/TE = 1800/40 ms) with a matrix = 160/128/64, repetitions = 2, field-of-view = 32/25.6/12.8 cm.

### Image analyses

Dynamic PET-images were co-registered with the anatomical MRI using a sum image of the first eight minutes of the PET scan to determine transformation parameters. Automated registration was achieved using mutual information algorithm implemented in statistical parametric mapping software (SPM12, Wellcome Institute, London, UK). Next, non-linear image registration implemented in SPM12 was used to find a mapping between individual anatomical MR-images and a Sprague Dawley rat brain template.^30^ Subsequently, dynamic PET-images were spatially aligned and resliced to match the atlas coordinates using the same mappings. Voxel size of the atlas was resampled to 0.1 mm × 0.1 mm × 0.1 mm in favor of reasonable data matrix size relative to the inherent spatial resolution of PET imaging data. Time-activity course (TAC) data were extracted from all anatomical regions (n = 222) defined in Waxholm space atlas of the Sprague Dawley rat brain version 4 (https://www.nitrc.org/projects/whs-sd-atlas).

### PET pharmacokinetic analysis

Administration of amphetamine was expected to lead to displacement of [^11^C]raclopride from its D2DR binding sites according to the competition principle.^17^ The primary variable of interest from the PET pharmacokinetic analysis was D2DR occupancy time-course data following in-scan amphetamine injections. To that end, lp-ntPET was used to estimate dynamic binding potential (BP_ND_) relative to non-displaceable binding in the cerebellum from ROI-wise TACs.^25–27,31^ In brief, grey matter voxels of the cerebellum was used as a reference region, as it is largely devoid of specific D2DR binding. A library of 180 gamma functions was generated^32^ with varying onset and peak times as well as growth and decay rates. This library was used as basis functions, and fitted as an additional variable in the multilinear reference tissue model.^33^ The onset time for ligand displacement was allowed to vary between 25 and 29 minutes in the model fitting. Models accounting for no displacement (base model) and 180 different shapes of displacement were assessed using Akaike information criteria (AIC), and the best fitting function was interpreted to reflect the most likely trajectory of [^11^C]raclopride displacement following the amphetamine/saline injections. The basis function approach permits analysis of inter-rat and inter-regional differences in [^11^C]raclopride displacement, which is appropriate given the unknown dynamics of DA release. Model fits were conducted for each animal and ROI independently, to generate a data set of regional dynamic BP_ND_ curves for subsequent statistical analysis. In the next step, the dynamic BP_ND_ estimate curves were transformed to dynamic D2DR occupancy profiles:

(1) $$\mathrm{Occupancy}\;\left(t;\%\right)=\frac{{\mathrm{pre}\;BP}_{ND}-{\mathrm{post}\;BP}_{ND}(t)}{{\mathrm{pre}\;BP}_{ND}}\times 100$$

where *pre BP_ND_* is the static estimate of [^11^C]raclopride binding prior to injection (0 – 25 minutes), and *post BP_ND_(t)* is the dynamic [^11^C]raclopride binding estimate after the injection (*t* = 25 – 60 minutes) derived using lp-ntPET.

### Statistical analysis

**Figure 1.**
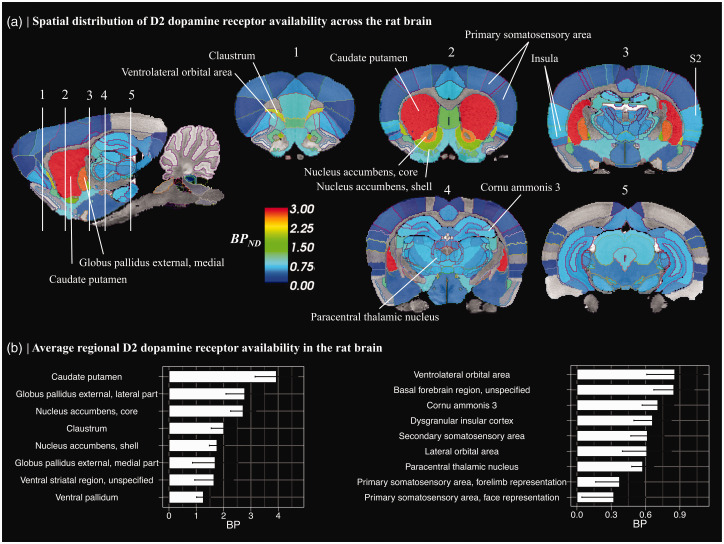
(a) Spatial distribution of [^11^C]raclopride binding potential (BP_ND_). Average of control animals’ (n = 6 scans) ROI-wise BP_ND_ values were overlayed on standard Sprague Dawley rat T2* MR-image. Lines depict region-of-interest borders of the anatomical atlas. BP_ND_ was calculated in regions of gray matter, with cerebellum serving as a reference region. Regions where BP_ND_ > 0.1 are shown. S2 = secondary somatosensory area and (b) Regional average (±95%CI) for [^11^C]raclopride BP_ND_ values in selected brain regions.

**Figure 2.**
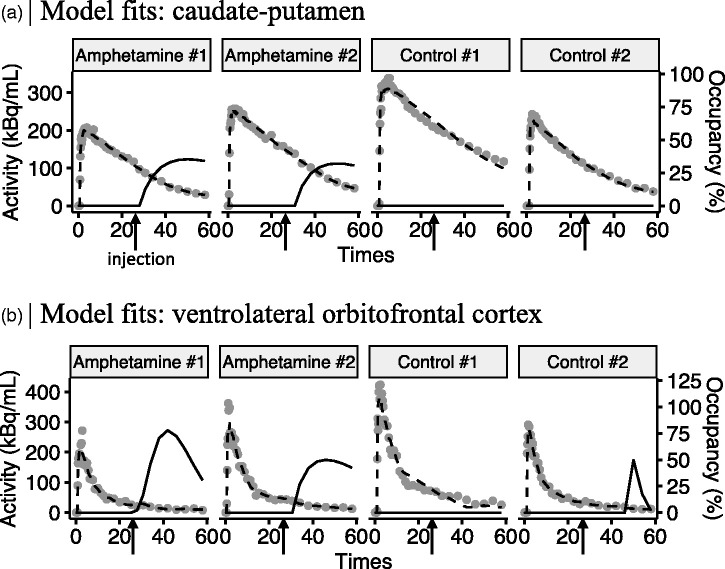
Representative pharmacokinetic model fits and corresponding dopamine occupancy profiles for caudate-putamen (a) and ventrolateral orbitofrontal cortex and (b) in 2 animals receiving amphetamine, and 2 animals injected with saline (control). Gray dots represent average radioactivity in the caudate-putamen, and dashed lines the lp-ntPET model fits. Occupancy (%) curves were calculated relative to a temporally stationary [^11^C]raclopride BP_ND_ estimate during first 25 minutes of the scan (solid black lines). Amphetamine or saline injections were given at 25 minutes after [^11^C]raclopride. Hence, occupancy (%) reflects how much specifically bound [^11^C]raclopride displacement was induced by each injection.

The main aims of the statistical analyses were: 1) to evaluate interregional differences in [^11^C]raclopride BP_ND_ across the brain (Figure 1), 2) to map regions showing statistically significant differences in [^11^C]raclopride occupancy profiles following amphetamine, as compared to saline, and 3) to characterize amphetamine-induced temporal trajectories of [^11^C]raclopride displacement. Significance level was set to *p* < 0.05 for all tests, and Shapiro-Wilk test of normality was applied when applicable. In all statistical analyses, repeated observations obtained from one individual were treated as independent observations. The rationale was to retain statistical power, while reducing the number of animals included in the study, under the assumption that the animals scanned twice did not systematically differ from the rest. This assumption was tested using Student’s t-test for a between-group difference (single/double scans) in baseline BP_ND_ (Shapiro-Wilks test FDR-adjusted p ≥ 0.11) and using Wilcoxon test in maximum occupancy after amphetamine (Shapiro-Wilks test indicated deviations from normal distribution in several ROIs). Results show that the animals that provided multiple observations did not differ from the rest in terms of pre-amphetamine BP_ND_ or maximum amphetamine-induced occupancy. This was confirmed for all ROIs (FDR-adjusted p ≥ 0.17 and p ≥ 0.97, respectively).

Interregional differences in BP_ND_ levels (aim 1) were investigated in the control group, exclusively (n = 6). A likely difference in the mean BP_ND_s of two regions was inferred when non-overlapping mean ± 95% confidence intervals (CI) between the two regions was observed. For aim 2, hierarchical general additive models (HGAM) were set up to assess for a group (amphetamine/saline) difference in non-linear occupancy profiles by having group membership as a random effect.^34^ Package mgcv (version 1.8) implemented in R (R Foundation for Statistical Computing, Vienna, Austria) was used for HGAM analysis.^35^ Specifically, for each region, temporal occupancy profiles in equation (1) were entered into GAM to allow modeling of group-average non-linear occupancy profiles. Base model (model 1) made no distinction between animals receiving amphetamine or saline. Improvement in model fit by inclusion of the random effect of group (amphetamine/saline, model 2) was interpreted as an indication of group difference in occupancy. Models were compared in terms of AIC and using chi-square test for unbiased calculation of *p*-values. Finally, to characterize amphetamine-induced temporal trajectories of [^11^C]raclopride displacement (aim 3), regional mean occupancy profiles (equation (1)) were generated using general additive models (GAM), focusing on the regions identified in step 2. Specifically, model-based predictions (forward modeling) were used to attain noise-suppressed mean occupancy profiles across the scans and ROIs forming anatomically and functionally continuous brain regions. Furthermore, 95% CI of the mean effect and derivatives^34^ of the temporal trajectories for the GAM predictions were calculated. Derivatives of temporal occupancy trajectories were used for identification of critical time intervals of displacement-rate variation. Furthermore, amphetamine-induced occupancy profiles relative to saline condition were calculated to mitigate the contribution of modeling errors using lp-ntPET. To that end, mean GAM-predicted occupancy profile of saline group was subtracted from that of amphetamine group, and 95% CI was calculated using standard error of the sum:

$$se=\sqrt{{se}_{\mathrm{Amphetamine}}^{2}+{se}_{\mathrm{Saline}}^{2}}.$$

## Results

### Interregional differences in [^11^C]raclopride binding potential in the rat brain

Spatial distribution and rank order of select regional [^11^C]raclopride BP_ND_ estimates are presented in Figure 1. As expected, highest bindings were observed in the basal ganglia, including caudate-putamen, ventral striatum, and globus pallidus (Figure 1(a) and (b)). Binding in the thalamus, hippocampal complex, and cortical regions was approximately five-fold lower as compared to the striatum (Figure 1(b)). Interregional differences in mean BP_ND_ were observed in extrastriatal regions, with highest cortical binding in the ventral orbital cortices and basal forebrain, followed by insular cortex, secondary somatosensory area, hippocampal complex and thalamus, and markedly lower binding in the primary somatosensory areas (Figure 1(b)). Binding in the primary visual cortex, a region analogous to the primate occipital cortex did not exceed 0.1 (Figure 1(a)) and did not statistically differ from zero (t-test, p = 0.062), thus, our results are in accordance with past studies showing negligible [^11^C]raclopride binding in the occipital cortex.^36^

### Interregional differences of amphetamine-induced [^11^C]raclopride displacement

**Figure 3.**
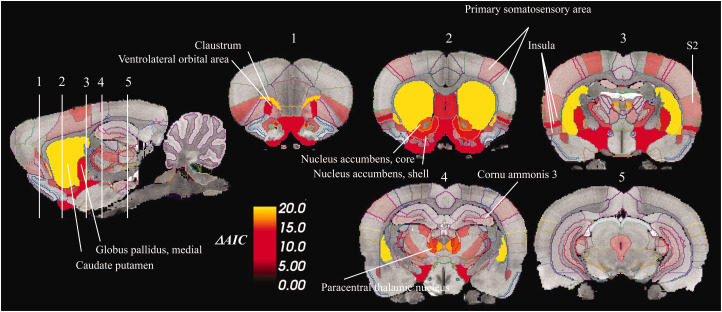
Spatial distribution of model comparison statistics (ΔAIC) for identification of regions where saline- versus amphetamine-induced [^11^C]raclopride displacement were significantly different. Difference in Akaike information criteria (ΔAIC) depict differences in GAM fits, reflecting the distance between the two groups in terms of the effects of respective injections. Models were additionally compared using chi-square test for unbiased calculation of *p*-values. Regions with *p*-values <0.05, following adjustment for false discovery rate, are shown.

Next, pharmacokinetic modeling of [^11^C]raclopride signal using lp-ntPET was conducted to estimate D2DR occupancy profiles following amphetamine and saline injections. As expected, amphetamine injections were associated with positive occupancy profiles, and no consistent changes in occupancy following saline injections (Figure 2). Amphetamine was associated with distinct [^11^C]raclopride responses compared to saline in several striatal as well as extrastriatal regions (Table 1); amounting to 49 ROIs, or 20% of all examined ROIs. Largest AIC differences – reflecting saline/amphetamine group difference in HGAM – were found in the basal ganglia and parts of thalamus, followed by insula, orbitofrontal cortex and secondary somatosensory area (Table 1, Figure 3). The whole-brain map of AIC differences (Figure 3) highlights pronounced interregional variation in the effects of amphetamine relative to saline.

**Table 1.** Summary of model-comparison statistics for identification of regions where the temporal profile of specific [^11^C]raclopride binding was altered differently after amphetamine as compared to saline injections.

Region	AIC (base model)	AIC (group model)	ΔAIC	*p* (FDR-adjusted)
Caudate putamen	1655.21	1604.45	−50.76	<10^−10^
Claustrum	1653.79	1606.91	−46.88	<10^−5^
Mediodorsal thalamic nucleus, medial part	2049.85	2007.46	−42.39	<10^−5^
Paracentral thalamic nucleus	1890.80	1856.21	−34.59	<10^−5^
Mediodorsal thalamic nucleus, lateral part	1933.54	1899.72	−33.83	<10^−5^
Nucleus accumbens, core	1802.86	1769.81	−33.05	<10^−5^
Nucleus accumbens, shell	1819.09	1790.67	−28.42	<10^−5^
Globus pallidus external, lateral part	1729.39	1701.39	−28.00	<10^−5^
Ventrolateral orbital area	1925.95	1899.19	−26.76	<10^−5^
Basal forebrain region, unspecified	1672.15	1645.92	−26.23	<10^−5^
Dysgranular insular cortex	1410.53	1392.94	−17.59	0.00039
Septal region	1642.25	1625.37	−16.88	0.00014
Mediodorsal thalamic nucleus, central part	2026.72	2011.11	−15.61	0.00135
Ventral striatal region, unspecified	1822.25	1807.17	−15.08	0.00016
Reticular (pre)thalamic nucleus, auditory segment	1930.07	1915.71	−14.35	0.00012
Reticular (pre)thalamic nucleus, unspecified	1640.66	1627.99	−12.68	0.00107
Endopiriform nucleus	1672.50	1661.70	−10.81	0.00093
Laterodorsal thalamic nucleus, dorsomedial part	1991.22	1980.51	−10.71	0.00045
Lateral posterior thalamic nucleus, mediorostral	1880.64	1870.55	−10.09	0.00049
Primary somatosensory area, hindlimb	1960.01	1950.35	−9.66	0.00075
Ventral pallidum	1837.00	1827.64	−9.37	0.00135
Agranular insular cortex dorsal area	1574.58	1565.39	−9.19	0.00377
Ventral posteromedial thalamic nucleus	1803.10	1795.56	−7.54	0.00204
Granular insular cortex	1587.86	1581.08	−6.78	0.00459
Secondary somatosensory area	1619.27	1612.79	−6.48	0.00459
Xiphoid thalamic nucleus	2056.51	2050.67	−5.84	0.00204
Periaqueductal gray	1774.30	1768.69	−5.61	0.00204
Zona incerta, dorsal part	1853.92	1849.06	−4.87	0.00594
supraoptic decussation	2001.97	1997.11	−4.86	0.00377
Ventral orbital area	1886.45	1881.65	−4.80	0.00459
Anteromedial thalamic nucleus	1938.43	1933.65	−4.78	0.00459
Globus pallidus external, medial part	1785.14	1780.69	−4.45	0.00795
Zona incerta, ventral part	1953.27	1949.41	−3.86	0.00681
Dorsal cochlear nucleus, deep core	2208.89	2205.34	−3.55	0.00459
Medial geniculate body, dorsal division	2001.63	1998.18	−3.45	0.00858
Subgeniculate nucleus	1947.70	1944.27	−3.43	0.00858
alveus of the hippocampus	1893.56	1890.16	−3.40	0.00858
Lateral orbital area	1787.14	1783.82	−3.32	0.01050
Agranular insular cortex, posterior area	1709.61	1706.57	−3.04	0.01300
Primary somatosensory area, forelimb	1822.07	1819.06	−3.01	0.01114
internal medullary lamina	1848.09	1845.19	−2.91	0.01114
Dorsal cochlear nucleus, molecular layer	2222.44	2219.60	−2.84	0.00459
Primary somatosensory area, dysgranular zone	1828.59	1825.75	−2.83	0.01050
Primary somatosensory area, barrel field	1809.92	1807.54	−2.38	0.01251
Ventrolateral thalamic nucleus	1854.70	1852.36	−2.34	0.01251
genu of the facial nerve	2142.46	2140.21	−2.25	0.00681
Posterior intralaminar nucleus	1989.92	1987.69	−2.23	0.00681
Pregeniculate nucleus	1901.21	1899.03	−2.18	0.01198
Cornu ammonis 3	1704.50	1702.37	−2.13	0.01300

Base model refers to a general additive model (GAM) of occupancy profiles omitting the specification of saline/amphetamine group, whereas the group model includes the group membership as a random effect. Difference in Akaike information criteria (ΔAIC) depict differences in GAM fits, reflecting the distance between the two groups in terms of the effects of respective injections. Models were additionally compared using chi-square test for unbiased calculation of *p*-values. Regions showing false discovery rate (FDR) adjusted *p* < 0.05 are reported. *N_amphetamine_* = 14, *N_salin_*_e_ = 6.

### Temporal profiles of amphetamine-induced [^11^C]raclopride displacement

Regions showing the most clear change in occupancy following amphetamine administration (parts of basal ganglia and thalamus, followed by insula, orbitofrontal cortex and secondary somatosensory area (Table 1; Figure 3), were further analyzed in terms of temporal DA occupancy profiles.

**Figure 4.**
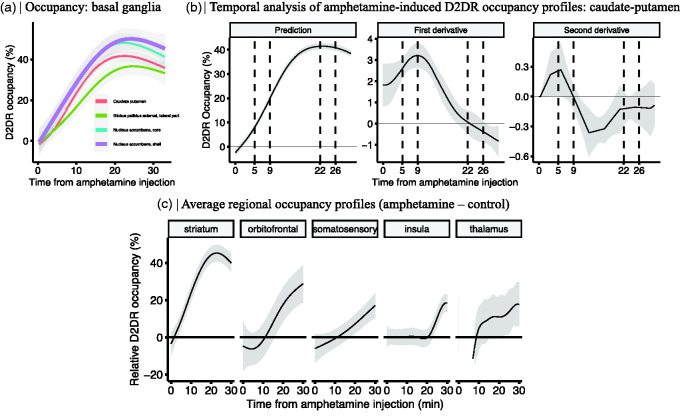
(a) Regional differences in the occupancy profiles of the basal ganglia. (b) Derivatives of the predicted occupancy time profiles of the caudate-putamen and (c) Striatal and extrastriatal amphetamine-induced D2R occupancy (%) time profiles (n = 14) relative to a control group (n = 6). Shaded area represents 95% CI.

First, we focused on amphetamine-induced occupancy time-courses in subregions of the basal ganglia (caudate-putamen, nucleus accumbens, and globus pallidus), i.e. the main targets of dopaminergic innervation. The shapes of occupancy time-courses were not markedly different across these regions (Figure 4(a)), suggesting a fairly similar temporal trajectory of amphetamine across these nuclei. However, the peak occupancies varied from ∼45% in the nucleus accumbens, to ∼40% in the caudate-putamen, and ∼35% in the globus pallidus. Further data-driven analysis using the GAM-derivatives revealed significant fluctuations in the occupancy rate (Figure 4(b)). Peak rate of occupancy increase was detected at 9 minutes after amphetamine injection (Figure 4(b); first derivative), while peak acceleration was seen at approximately 5 minutes after the injection (Figure 4(b); second derivative). Peak receptor occupancy occurred at approximately 22 minutes after the injection (Figure 4(b); prediction), and significant decline in occupancy was observed at approximately 26 minutes (Figure 4(b); first derivative shows significant negative trend).

Next, we considered extrastriatal regions showing amphetamine-induced change in occupancy and compared these with the striatal occupancy profiles. In order to mitigate the effects of statistical noise and to summarize the results across the brain, the estimates of occupancy (Table 1) were pooled across ROIs to form anatomical clusters. Specifically, five anatomical regions were considered, constituting the striatum (caudate-putamen, nucleus accumbens), orbitofrontal cortex, somatosensory cortex, insula and thalamus, each consisting of three to twelve subregions. Shape differences in the regional occupancy profiles were estimated by extracting the predicted occupancy profile of the control group (6 scans) from that of the amphetamine group (14 scans; Figure 4(c)). The relative occupancy time profiles revealed marked interregional differences in the timing as well as magnitude of amphetamine-induced [^11^C]raclopride displacement. Notably, the peak magnitude of occupancy was higher and increase in occupancy was more rapid in the striatum, in comparison to extrastriatal regions (Figure 4(c)). While the narrowest 95% CI was found for the striatum, select extrastriatal regions also exhibited reliable amphetamine-induced time-resolved occupancies relative to the control condition.

## Discussion

The present work shows interregional differences in amphetamine-induced DA release, as assessed using PET and [^11^C]raclopride. In line with previous findings in non-human primates,^28^ there was a ventro-dorsal gradient of DA release in the striatum, with significantly higher D2DR occupancy in the nucleus accumbens as compared to caudate-putamen. Furthermore, timing of peak striatal D2DR occupancy was remarkably similar to previously reported amphetamine-induced DA concentration peak using microdialysis.^1^ Apart from the striatum, DA release was also found in several extrastriatal regions, including thalamus, insula, orbitofrontal cortex and secondary somatosensory area. Previously, D2DR mRNA quantifications indeed show that D2DR are expressed across the cortex in rats, and particularly in prefrontal regions.^37–39^ In agreement, we show higher [^11^C]raclopride binding in cortical regions, as compared to the reference region (where D2DR expression is close to zero). In concordance with the present work, prior human studies have found DA release following amphetamine in the orbitofrontal cortex and insula.^40,41^ In terms of temporal profiles for occupancy changes, striatum and globus pallidus were characterized by earlier onset and higher peak magnitude of occupancy, as compared to extrastriatal regions. This is expected given prior findings of large differences in striatal and cortical DA release following a comparable dose of d-amphetamine, peaking at about 1500% versus 300-600% above baseline, respectively.^19,20^ Together these findings suggest reliable detection of amphetamine-induced DA release using [^11^C]raclopride in striatal as well as in several extrastriatal regions. Nevertheless, given the low extrastriatal occupancies relative to methodological noise (Figure 4(c)), it is advisable to proceed with caution in experimental settings in which a group difference is desired outside the striatum. The present proof-of-concept study exploited a maximum contrast between groups (amphetamine/saline), whereas more modest differences would likely require larger groups and/or regularization of methodological noise to detect significant effects.

Drug addiction is associated with altered function of the orbitofrontal cortex.^42^ Volumetric changes for orbitofrontal cortex, insula, striatum, and medial temporal lobe follow chronic use of cocaine and amphetamine.^43^ Furthermore, activation of insula and striatum during reward-based tasks are associated with liking of amphetamine.^44^ Here, we show that DA activity is altered in these regions following amphetamine intake, with the exception for medial temporal lobe. The absence of occupancy change in medial temporal lobe was partly surprising given significant amphetamine-induced DA level elevations in past microdialysis work.^19^ It may be the case that DA release is lower in medial temporal lobe, as compared to prefrontal regions, and thus displacement is not detectable. Pum and colleagues indeed reported an elevation of approximately 300% above baseline at 40 minutes in parts of the medial temporal lobe,^19^ while twice as high (∼600%) DA release was observed in the medial prefrontal cortex, not far from the ventrolateral orbital cluster observed here. The combination of low signal-to-noise ratio of [^11^C]raclopride with the well-established ceiling effects to [^11^C]raclopride displacement by endogenous DA^17,45^ may have prohibited the detection of putative DA release in some extrastriatal regions. Specifically, based on investigations including rodents, it has been postulated that approximately 50% of D2DRs that bind [^11^C]raclopride are in low-affinity state towards endogenous DA, and therefore, may not contribute to the pool of receptors under active competition.^17,45^ Here, this ceiling effect likely contributed to the striatal D2DR occupancy data plateauing at approximately 50%, despite up to 1500% increase in DA concentration following a similar dose of amphetamine as employed here has been reported.^1^ Furthermore, it has been pointed out that while specific [^11^C]raclopride binding is detectable outside of the striatum, cerebellum may not be optimal as reference region for low-density D2DR regions in occupancy studies.^46^ This since the D2DR levels are low, but not completely absent, in the cerebellum.^8^

[^11^C]raclopride has been used as a radioligand for over 30 years. When introduced, it was deemed suitable for striatal quantifications only, due to its low signal-to-noise ratio.^8^ A growing amount of evidence now suggests that, although extrastriatal D2DR levels are low, they are detectable with [^11^C]raclopride.^46^ To exemplify, test-retest reliability is good-to-excellent not only for striatal regions, but also for example for thalamus, and frontal and temporal cortices.^2,3^ Furthermore, extrastriatal D2DR levels determined with [^11^C]raclopride map well with high-affinity DRD2 ligand [^18^F]fallypride.^3,11^ Here, evidence of amphetamine-induced extrastriatal [^11^C]raclopride displacement was found for some extrastriatal regions, which is not surprising given variability in extrastriatal DAT concentration across brain regions.^47^ In regions with low DAT concentration, such as the frontal cortex, noradrenaline transporters may have a significant role in DA clearance.^48^ The striatal and cortical D2DR occupancy trajectories differed not merely by a clear level difference, but also in terms of the rate of accumulation. This finding is particularly well-aligned with past non-human primate research, showing a striking difference in magnitude and timing between striatal and cortical amphetamine-induced DA release as assessed using microdialysis.^20^ The present finding thus support previous observations of greater magnitude and more rapid displacement of [^11^C]raclopride in the striatum, which is due to higher concentration of DA nerve terminals and, therefore, more capacity for amphetamine-induced reverse-transport of DA^21^. That said, future studies should investigate occupancy profiles beyond thirty minutes from amphetamine administration, in order to capture the peak in extrastriatal occupancy. In contrast to prior studies, whole-brain coverage and temporal characterization of amphetamine-induced DA release was achieved by regional, time-sensitive analysis of [^11^C]raclopride signal.

Effects related to limited spatial resolution of PET scanning may have affected the outcome of the present analysis. Specifically, the remarkably high [^11^C]raclopride BP_ND_ in the striatum relative to the background renders the signal in the neighboring regions susceptible to the so-called spillover effect.^49^ Therefore, it is conceivable that spillover effect may have contributed to the high BP_ND_ estimates in the secondary somatosensory area and insula, but it is harder to reconcile why other close-by regions such as primary motor area did not show high BP_ND_; as the spillover effect should be fairly similar across regions close to striatum.^49^ Moreover, the majority of past PET studies have used a difference between static measures of pre- and post-amphetamine BP_ND_. Here, instead, amphetamine was administered during PET scanning and the dynamics of [^11^C]raclopride displacement were assessed with the help of temporally sensitive PET pharmacokinetic modeling (lp-ntPET).^24–27^ The dynamic approach is advantageous to static measures, given that static measures depend on the time of measurement (relative to amphetamine administration), and that amphetamine-induced DA release exhibit markedly different temporal dynamics across the brain.^19,20^ Therefore, the current study presents an approach that might be crucial for simultaneous detection of amphetamine-induced [^11^C]raclopride displacement in the striatum and extrastriatal regions. Notably, the present study presents cortical [^11^C]raclopride binding potential values that are approximately 15% of those in the striatum, as compared to 6%–8% reported in past high-resolution human studies.^2,7^ This potential species difference in the strength of cortical [^11^C]raclopride signal should be considered when translating the present findings to human imaging.

In conclusion, the present findings suggest feasibility of single-scan [^11^C]raclopride competition study for whole-brain mapping of amphetamine-induced DA release in the rat brain. Translation of a similar technique to human imaging studies appears possible, given a growing body of evidence that not only striatal but also extrastriatal displacement of [^11^C]raclopride follows from pharmacological as well as non-pharmacological stimulation of DA release.^7,21–23^

## References

[1] Heal DJ Smith SL Gosden J , et al. Amphetamine, past and present – a pharmacological and clinical perspective. J Psychopharmacol 2013; 27: 479–496. 23539642 10.1177/0269881113482532 PMC3666194

[2] Alakurtti K Johansson JJ Joutsa J , et al. Long-term test–retest reliability of striatal and extrastriatal dopamine D2/3 receptor binding: study with [^11^C]raclopride and high-resolution PET. J Cereb Blood Flow Metab 2015; 35: 1199–1205. 25853904 10.1038/jcbfm.2015.53 PMC4640276

[3] Karalija N Jonassson L Johansson J , et al. High long-term test–retest reliability for extrastriatal ^11^C-raclopride binding in healthy older adults. J Cereb Blood Flow Metab 2020; 40: 1859–1868. 31506011 10.1177/0271678X19874770 PMC7446562

[4] Ginovart N. Imaging the dopamine system with in vivo [^11^C]raclopride displacement studies: understanding the true mechanism. Mol Imaging Biol 2005; 7: 45–52. 15912275 10.1007/s11307-005-0932-0

[5] Bäckman L Nyberg L Soveri A , et al. Effects of working-memory training on striatal dopamine release. Science 2011; 333: 718. 21817043 10.1126/science.1204978

[6] Egerton A Mehta MA Montgomery AJ , et al. The dopaminergic basis of human behaviors: a review of molecular imaging studies. Neurosci Biobehav Rev 2009; 33: 1109–1132. 19481108 10.1016/j.neubiorev.2009.05.005 PMC3797507

[bibr7-0271678X231210128] Svensson JE Schain M Plavén-Sigray P , et al. Validity and reliability of extrastriatal [^11^C]raclopride binding quantification in the living human brain. Neuroimage 2019; 202: 116143. 31473354 10.1016/j.neuroimage.2019.116143

[8] Farde L Pauli S Hall H , et al. Stereoselective binding of ^11^C-raclopride in living human brain – a search for extrastriatal central D2-dopamine receptors by PET. Psychopharmacology (Berl) 1988; 94: 471–478. 3131792 10.1007/BF00212840

[9] Gertler J Tollefson S Jordan R , et al. Failure to detect amphetamine-induced dopamine release in the cortex with [^11^ C]FLB 457 positron emission tomography (PET): methodological considerations. Synapse 2018; 72: e22037. 29876970 10.1002/syn.22037 PMC6230264

[10] Narendran R Frankle WG Mason NS , et al. Positron emission tomography imaging of amphetamine-induced dopamine release in the human cortex: a comparative evaluation of the high affinity dopamine D2/3 radiotracers [^11^C]FLB 457 and [^11^C]fallypride. Synapse 2009; 63: 447–461. 19217025 10.1002/syn.20628

[11] Papenberg G Jonasson L Karalija N , et al. Mapping the landscape of human dopamine D2/3 receptors with [^11^C]raclopride. Brain Struct Funct 2019; 224: 2871–2882. 31444615 10.1007/s00429-019-01938-1 PMC6778542

[12] Karalija N Johansson J Papenberg G , et al. Longitudinal dopamine D2 receptor changes and cerebrovascular health in aging. Neurology 2022; 99: e1278–e1289. 35790424 10.1212/WNL.0000000000200891 PMC9576296

[13] Wang GJ Volkow ND Fowler JS , et al. Age associated decrements in dopamine D2 receptors in thalamus and in temporal insula of human subjects. Life Sci 1996; 59: PL31–35. 8684263 10.1016/0024-3205(96)00262-7

[14] Salami A Andersson M Papenberg G , et al. Dopamine D 2/3 binding potential modulates neural signatures of working memory in a Load-Dependent fashion. J Neurosci 2019; 39: 537–547. 30478031 10.1523/JNEUROSCI.1493-18.2018 PMC6335744

[bibr15-0271678X231210128] Köhncke Y Papenberg G Jonasson L , et al. Self-rated intensity of habitual physical activities is positively associated with dopamine D 2/3 receptor availability and cognition. Neuroimage 2018; 181: 605–616. 30041059 10.1016/j.neuroimage.2018.07.036

[16] Nyberg L Karalija N Salami A , et al. Dopamine D2 receptor availability is linked to hippocampal–caudate functional connectivity and episodic memory. Proc Natl Acad Sci U S A 2016; 113: 7918–7923. 27339132 10.1073/pnas.1606309113 PMC4948341

[17] Laruelle M. Imaging synaptic neurotransmission with *in vivo* binding competition techniques: a critical review. J Cereb Blood Flow Metab 2000; 20: 423–451. 10724107 10.1097/00004647-200003000-00001

[18] Sulzer D Chen TK Lau YY , et al. Amphetamine redistributes dopamine from synaptic vesicles to the cytosol and promotes reverse transport. J Neurosci 1995; 15: 4102–4108. 7751968 10.1523/JNEUROSCI.15-05-04102.1995 PMC6578196

[19] Pum M Carey RJ Huston JP , et al. Dissociating effects of cocaine and d-amphetamine on dopamine and serotonin in the perirhinal, entorhinal, and prefrontal cortex of freely moving rats. Psychopharmacology (Berl) 2007; 193: 375–390. 17468969 10.1007/s00213-007-0791-2

[20] Jedema HP Narendran R Bradberry CW. Amphetamine-induced release of dopamine in primate prefrontal cortex and striatum: Striking differences in magnitude and timecourse. J Neurochem 2014; 130: 490–497. 24749782 10.1111/jnc.12743 PMC4126881

[21] Volkow ND Wang G-J Tomasi D , et al. Methylphenidate-elicited dopamine increases in ventral striatum are associated with long-term symptom improvement in adults with attention deficit hyperactivity disorder. J Neurosci 2012; 32: 841–849. 22262882 10.1523/JNEUROSCI.4461-11.2012 PMC3350870

[bibr22-0271678X231210128] Stokes PRA Egerton A Watson B , et al. Significant decreases in frontal and temporal [^11^C]-raclopride binding after THC challenge. Neuroimage 2010; 52: 1521–1527. 20451621 10.1016/j.neuroimage.2010.04.274

[23] Thanarajah SE Backes H DiFeliceantonio AG , et al. Food intake recruits orosensory and post-ingestive dopaminergic circuits to affect eating desire in humans. Cell Metab 2019; 29: 695–706.e4. 30595479 10.1016/j.cmet.2018.12.006

[24] Alpert NM Badgaiyan RD Livni E , et al. A novel method for noninvasive detection of neuromodulatory changes in specific neurotransmitter systems. Neuroimage 2003; 19: 1049–1060. 12880831 10.1016/s1053-8119(03)00186-1

[bibr25-0271678X231210128] Normandin MD Schiffer WK Morris ED. A linear model for estimation of neurotransmitter response profiles from dynamic PET data. Neuroimage 2012; 59: 2689–2699. 21767654 10.1016/j.neuroimage.2011.07.002 PMC3702051

[bibr26-0271678X231210128] Johansson J Hirvonen J Lovró Z , et al. Intranasal naloxone rapidly occupies brain mu-opioid receptors in human subjects. Neuropsychopharmacology 2019; 44: 1667–1673. 30867551 10.1038/s41386-019-0368-x PMC6785104

[27] Sander CY Hooker JM Catana C , et al. Neurovascular coupling to D2/D3 dopamine receptor occupancy using simultaneous PET/functional MRI. Proc Natl Acad Sci U S A 2013; 110: 11169–11174. 23723346 10.1073/pnas.1220512110 PMC3703969

[28] Drevets WC Price JC Kupfer DJ , et al. PET measures of amphetamine-induced dopamine release in ventral versus dorsal striatum. Neuropsychopharmacology 1999; 21: 694–709. 10633475 10.1016/S0893-133X(99)00079-2

[29] Hall H Sedvall G Magnusson O , et al. Distribution of D1- and D2-dopamine receptors, and dopamine and its metabolites in the human brain. Neuropsychopharmacology 1994; 11: 245–256. 7531978 10.1038/sj.npp.1380111

[30] Papp EA Leergaard TB Calabrese E , et al. Waxholm space atlas of the sprague dawley rat brain. Neuroimage 2014; 97: 374–386. 24726336 10.1016/j.neuroimage.2014.04.001 PMC4160085

[31] Grill F Johansson J Axelsson J , et al. Dissecting motor and cognitive component processes of a finger-tapping task with hybrid dopamine positron emission tomography and functional magnetic resonance imaging. Front Hum Neuro 2021; 15: 1–9. 10.3389/fnhum.2021.733091 PMC8667474 34912200

[32] Madsen MT. A simplified formulation of the gamma variate function. Phys Med Biol 1992; 37: 1597–1600.

[33] Ichise M Liow JS Lu JQ , et al. Linearized reference tissue parametric imaging methods: application to [^11^C]DASB positron emission tomography studies of the serotonin transporter in human brain. J Cereb Blood Flow Metab 2003; 23: 1096–1112. 12973026 10.1097/01.WCB.0000085441.37552.CA

[34] Pedersen EJ Miller DL Simpson GL , et al. Hierarchical generalized additive models in ecology: an introduction with mgcv. PeerJ 2019; 2019; 7: e6876. 31179172 10.7717/peerj.6876 PMC6542350

[35] Wood SN. Generalized additive models: an introduction with R, second edition. Boca Raton: Chapman and Hall/CRC, 2017.

[36] Lidow MS Goldman-Rakic PS Rakic P , et al. Dopamine D2 receptors in the cerebral cortex: distribution and pharmacological characterization with [3H]raclopride. Proc Natl Acad Sci U S A 1989; 86: 6412–6416. 2548214 10.1073/pnas.86.16.6412 PMC297850

[37] Yu Q Liu YZ Zhu YB , et al. Genetic labeling reveals temporal and spatial expression pattern of D2 dopamine receptor in rat forebrain. Brain Struct Funct 2019; 224: 1035–1049. 30604007 10.1007/s00429-018-01824-2 PMC6499762

[bibr38-0271678X231210128] Weiner DM Levey AI Sunahara RK , et al. D1 and D2 dopamine receptor mRNA in rat brain. Proc Natl Acad Sci U S A 1991; 88: 1859–1863. 1825729 10.1073/pnas.88.5.1859 PMC51125

[39] Santana N Artigas F. Laminar and cellular distribution of monoamine receptors in rat medial prefrontal cortex. Front Neuroanat 2017; 11: 87–13. 29033796 10.3389/fnana.2017.00087 PMC5625028

[40] Cropley VL Innis RB Nathan PJ , et al. Small effect of dopamine release and no effect of dopamine depletion on [18F]fallypride binding in healthy humans. Synapse 2008; 62: 399–408. 18361438 10.1002/syn.20506

[41] Smith CT Dang LC Cowan RL , et al. Variability in paralimbic dopamine signaling correlates with subjective responses to d-amphetamine. Neuropharmacology 2016; 108: 394–402. 27174408 10.1016/j.neuropharm.2016.05.004 PMC4912942

[42] Schoenbaum G Shaham Y. The role of orbitofrontal cortex in drug addiction: a review of preclinical studies. Biol Psychiatry 2008; 63: 256–262. 17719014 10.1016/j.biopsych.2007.06.003 PMC2246020

[43] Mackey S Paulus M. Are there volumetric brain differences associated with the use of cocaine and amphetamine-type stimulants? Neurosci Biobehav Rev 2013; 37: 300–316. 23253945 10.1016/j.neubiorev.2012.12.003 PMC3604030

[44] Langenecker SA Kling LR Crane NA , et al. Anticipation of monetary reward in amygdala, insula, caudate are predictors of pleasure sensitivity to d-Amphetamine administration. Drug Alcohol Depend 2020; 206: 107725. 31757518 10.1016/j.drugalcdep.2019.107725 PMC6980714

[45] Caravaggio F Porco N Kim J , et al. Measuring amphetamine-induced dopamine release in humans: a comparative meta-analysis of [^11^C]-raclopride and [^11^C]-(+)-PHNO studies. Synapse 2021; 75: e22195–18. 33471400 10.1002/syn.22195

[46] Backes H. [^11^C]raclopride and extrastriatal binding to D2/3 receptors. Neuroimage 2020; 207: 116346. 31715255 10.1016/j.neuroimage.2019.116346

[47] Ito H Takahashi H Arakawa R , et al. Normal database of dopaminergic neurotransmission system in human brain measured by positron emission tomography. Neuroimage 2008; 39: 555–565. 17962043 10.1016/j.neuroimage.2007.09.011

[48] Morón JA Brockington A Wise RA , et al. Dopamine uptake through the norepinephrine transporter in brain regions with low levels of the dopamine transporter: evidence from knock-out mouse lines. J Neurosci 2002; 22: 389–395. 11784783 10.1523/JNEUROSCI.22-02-00389.2002 PMC6758674

[49] Lehnert W Gregoire MC Reilhac A , et al. Characterisation of partial volume effect and region-based correction in small animal positron emission tomography (PET) of the rat brain. Neuroimage 2012; 60: 2144–2157. 22387126 10.1016/j.neuroimage.2012.02.032

